# CM352 Reduces Brain Damage and Improves Functional Recovery in a Rat Model of Intracerebral Hemorrhage

**DOI:** 10.1161/JAHA.117.006042

**Published:** 2017-06-01

**Authors:** José A. Rodríguez, Tomás Sobrino, Esteban López‐Arias, Ana Ugarte, Juan A. Sánchez‐Arias, Alba Vieites‐Prado, Irene de Miguel, Julen Oyarzabal, José A. Páramo, Francisco Campos, Josune Orbe, José Castillo

**Affiliations:** ^1^ Atherosclerosis Research Laboratory Health Research Institute of Navarra‐IdiSNA University of Navarra Pamplona Spain; ^2^ Small Molecule Discovery Platform Molecular Therapeutics Program Center for Applied Medical Research Health Research Institute of Navarra‐IdiSNA University of Navarra Pamplona Spain; ^3^ Hematology Service Clínica Universidad de Navarra Health Research Institute of Navarra‐IdiSNA University of Navarra Pamplona Spain; ^4^ Clinical Neurosciences Research Laboratory Department of Neurology Hospital Clínico Universitario Health Research Institute of Santiago de Compostela Universidade de Santiago de Compostela Spain; ^5^ CIBER Cardiovascular Instituto de Salud Carlos III Madrid Spain

**Keywords:** brain, CM‐352, fibrinolysis, matrix metalloproteinases, hemorrhagic stroke, Intracranial Hemorrhage, Animal Models of Human Disease, Translational Studies, Pharmacology

## Abstract

**Background:**

Intracerebral hemorrhage (ICH) is an acute neurological disorder with high mortality and no effective treatment. In addition to the initial bleeding event, rebleeding and hematoma expansion are associated with poor outcome in these patients. We studied the effectiveness of the new antifibrinolytic agent CM352, a short‐half‐life matrix metalloproteinase inhibitor, for achieving early hemostasis and improving functional recovery in a rat model of collagenase‐induced ICH.

**Methods and Results:**

ICH was induced by striatal injection of collagenase, and 1 hour later, rats received an intravenous injection of saline (n=6) or CM352 (1 mg/kg, n=6). Hematoma (basal and after 3 and 24 hours) and lesion (14 days) volumes were quantified on T2‐weighted (T2) magnetic resonance images. Neurological and functional recovery was evaluated by using Bederson score and a cylinder test (basal, 24 hours, and 14 days). Early treatment (1 hour) with CM352 was efficient reducing hematoma expansion at 3 hours (*P*<0.01) and, more markedly, at 24 hours (*P*<0.01). Decreased bleeding after antifibrinolytic treatment was accompanied by reduced interleukin‐6 levels at 3 hours (*P*<0.05) and smaller lesion volume at 14 days (*P*<0.01). CM352 drastically reduced sensorimotor impairment (cylinder test) after ICH in rats at 24 hours (*P*<0.01) and 14 days (*P*<0.01). Similarly, it also attenuated neurological deficit (Bederson scale) at 24 hours (*P*<0.01) and 14 days (*P*<0.01). Interestingly, late (3 hours) CM352 administration also resulted in reduced lesion size and better functional outcome.

**Conclusions:**

CM352, a new antifibrinolytic agent and matrix metalloproteinase inhibitor, effectively prevented hematoma growth and reduced lesion size in ICH in association with improved functional and neurological recovery.

**Clinical Perspective**

**What Is New?**

- Dual antifibrinolytic and antiproteolytic treatment, with a fast‐acting short‐lived matrix metalloprotease (MMP) inhibitor, can effectively reduce hematoma expansion and brain damage in an experimental model of intracerebral hemorrhage.
- This treatment is associated with less inflammation together with better functional and neurological recovery.
- Treatment administration 1 hour (early) or 3 hours (late) after intracerebral hemorrhage onset achieves similar beneficial effects.

**What Are the Clinical Implications?**

- Our findings support the use of MMP inhibitors with antifibrinolytic properties for the pharmacological management of intracerebral hemorrhage, a life‐threatening condition with no proven treatment.
- This therapeutic approach could be particularly interesting in the setting of hemorrhagic transformation after thrombolytic treatment.

## Introduction

Although it comprises only ≈10% to 20% of all stroke cases, nontraumatic intracerebral hemorrhage (ICH) claims the most lives with 30% to 40% mortality, half of which occurs within 2 days of ICH onset.^1^ Hypertension being the most important risk factor, commonly used drugs such as anticoagulants, platelet inhibitors, and thrombolytics (tissue plasminogen activator) increase the risk of ICH.^2^, ^3^ In spite of the tremendous societal and economic burden in the aging population, there is no proven treatment for ICH, and its outcomes and prognosis have not improved significantly.^1^

ICH is triggered by vessel rupture and direct blood extravasation in the brain parenchyma, where hematoma mechanically disrupts neighboring blood vessels, increasing bleeding and hematoma growth, leading to ischemia and impaired neuronal function. Consequently, hematoma size and expansion are associated with poor outcome and early neurological deterioration after ICH.^4^, ^5^ Hematoma growth is also followed by a cascade of secondary events: neuroinflammation, edema formation, toxic effects of blood degradation products, leukocyte infiltration and activation, protease release, and further blood‐brain barrier disruption, which exacerbate the hemorrhagic lession.^6^

Because hematoma expansion is the only modifiable predictor of outcome after ICH, several medical therapies aimed at improving ICH by reducing it have been studied. To date aggressive blood pressure reduction,^7^ early neurosurgical intervention,^8^ and hemostatic therapy with recombinant active factor VII (rFVIIa)^9^ have failed to improve survival or functional outcome after ICH in unselected patients. Because hyperfibrinolysis is an important contributor to major bleeding events and has been associated with ICH,^10^ clinical trials are currently being conducted to assess the benefits of antifibrinolytic therapy (tranexamic acid [TXA]) to reduce hemorrhage and improve ICH outcome.^11^

Matrix metalloproteinases (MMPs) have been associated clinically and experimentally with extracellular matrix degradation triggered by inflammation after stroke. Particularly in ICH, MMPs have been proposed to play a main role in blood‐brain barrier disruption, brain edema, and neuronal death.^12^, ^13^ This evidence led to the testing of a variety of broad‐spectrum MMP inhibitors in experimental models of ICH. Among them, GM6001^13^ and minocycline^14^ achieved a significant reduction of secondary brain damage and functional improvement.

We and others have shown that MMPs cooperate with the fibrinolytic system to regulate thrombus dissolution: plasmin is capable of activating specific MMPs, which in turn participate in the dissolution of fibrin through direct targeting or interaction with other components of the fibrinolytic system.^15^, ^16^ A role of MMPs in hemorrhage is also supported by the association between increased levels of certain MMPs and prolonged bleeding in human and animal studies, whereas genetic deletion of MMPs prevented bleeding in experimental models.^17^, ^18^ Furthermore, our research group recently demonstrated that acute and short‐term MMP inhibition is an effective and safe therapeutic strategy for the prevention of bleeding. We used GM6001 as a proof of concept and developed CM352, a new short‐lived MMP inhibitor and highly efficient antifibrinolytic agent optimized for human blood that does not interfere with normal hemostatic function.^19^, ^20^

Therefore, the aim of this preclinical study was to determine whether CM352 treatment, through dual inhibition of fibrinolysis and proteolysis, can reduce hematoma expansion and improve functional and neurological recovery in a rat model of ICH induced by intraparenchymal injection of collagenase.

## Methods

4‐[4‐[[(3R)‐3‐(Hydroxycarbamoyl)‐8‐azaspiro[4.5]decan‐3‐yl]sulfonyl]phenoxy]‐N‐methylbenzamide (CM352) was synthesized and purified at WuXi AppTec (Tianjin, PR China).

### Animals

All experimental protocols were approved by the University Clinical Hospital of Santiago de Compostela Animal Care Committee and University of Navarra Ethics Committee for Animal Research, according to the European Union and Spanish rules (86/609/CEE, 2003/65/CE, 2010/63/EU, and RD53/2013). Male Sprague Dawley rats (Harlan Iberica, Barcelona, Spain) weighting 325 to 350 g were conditioned to the local animal facilities for at least 5 days before surgery. Rats were kept in day/night cycles of 12 hours at a mean temperature of 22±1°C and 60±5% humidity, watered and fed ad libitum.

### Pharmacokinetics Analysis of CM352 in Rat Samples

CM352 solutions were prepared by dissolving the solid in saline. A drug dosage of 1 mg/kg was administered as a single intravenous injection in the femoral vein. Blood was collected into citrated tubes, and plasma was obtained by 2‐step centrifugation: first at 600 g for 10 minutes and then at 18 000 g for 2 minutes. Plasma samples were stored at −80°C until analysis. CM352 was measured in plasma samples using a Xevo‐TQ MS triple quadrupole mass spectrometer with an electrospray ionization source and an Acquity UPLC (Waters, Manchester, UK) as previously reported.^20^ Briefly, after chromatographic separation, the compound was detected using multiple reaction monitoring. Quantification was achieved by external calibration using matrix‐matched standards prepared by adding the appropriate volume of diluted solutions of the compound (in methanol/water 50:50, v:v) to aliquots of 50 μL blank plasma. Pharmacokinetic parameters were obtained by fitting the blood concentration‐time data to a bicompartmental model with the WinNonlin software (Pharsight, Mountain View, CA).

### Whole Blood Clot Formation and Lysis Assay

Citrated blood samples from (n=4) rats (Sprague Dawley) and (n=16) mice (C57Bl6J, Harlan Iberica) were assayed with a ROTEM analyzer (Pentapharm GmbH, Munich, Germany) as previously described.^20^ In a prewarmed cuvette and holder we pipetted 1 μL tissue‐type plasminogen activator (150 000 U/mL), 20 μL Start‐tem reagent (Pentapharm GmbH), 20 μL In‐tem reagent (Pentapharm GmbH), 3 μL DMSO (SIGMA) or test compounds in DMSO, and 300 μL prewarmed citrated blood. The cup holder containing the sample mixture was placed immediately on the appropriate channel. Measurements were recorded for 60 minutes to allow clot formation and lysis, and results are expressed as effective concentration to delay lysis time by 50%.

### MMP Enzyme Activity Assays

The inhibitory effect of CM352 against MMP‐2, MMP‐7, MMP‐9, and MMP‐12 was tested at GenScript (http://www.genscript.com/), using GM6001 as reference compound. Protease stock solutions (10 mmol/L) were diluted with assay buffer to make final test solutions containing no more than 2.0% DMSO. The samples were dispensed into plates; substrate was added and read with PHERAstar PLUS (BMG Labtech, Ortenberg, Germany) plate reader.

### Collagenase Activity Assay

Enzymatic assay of collagenase was performed using FALGPA (Sigma‐Aldrich Corp, St. Louis, MO) as substrate, according to the manufacturer's recommendations. Briefly, proteolysis of 1 mmol/L FALGPA by 2 U/mL bacterial collagenase type VII (Sigma‐Aldrich Corp, St. Louis, MO) in reaction buffer (50 mmol/L Tricine; 10 mmol/L CaCl_2_; 400 mmol/L NaCl) was monitored spectrophotometrically (345 nm) for 5 minutes, in the presence of CM352, phenanthroline, or vehicle.

### ICH Rat Model

The ICH rat model was used as previously described.^21^ Anesthesia was maintained by inhalation of 4% sevoflurane in a N_2_O/O_2_ mixture (70/30), and body temperature was maintained at 37±0.5°C with a heating pad until animals completely recovered from anesthesia and displayed normal motor activity. Glucose levels were analyzed before surgery (mean 144.3±22.1 mg/dL). Rats were placed in a stereotaxic frame (Stoelting Co, Wood Dale, IL) under sevoflurane anesthesia. After drilling of a small burr hole, 1 μL of saline containing 0.2 U/μL bacterial collagenase type VII (Sigma‐Aldrich Corp, St. Louis, MO) was injected into the right striatum (0.6 mm anterior to bregma, −3.0 mm lateral and 5.5 mm depth) using a Hamilton syringe with a 30 G needle. Injections took place over a 10‐minute period, and the needle remained in place for additional 10 minutes before removal (1 mm/min). The burr hole was filled with bone wax (Ethicon, Somerville, NJ), and the scalp incision was closed with sutures.

### Experimental Groups

Two experimental groups (n=6) were designated: (1) a control group treated with saline (0.9% of NaCl) and (2) a CM352 group treated with 1 mg/kg CM352 in saline. The same experimental design and setting were used to assess the effect of late (3 hours) CM352 treatment. Required sample size was calculated from previous studies using the same model^21^ in order to be able to detect a 25% effect size on hematoma growth versus controls (2‐tailed t test). Six animals per group are required to detect this difference with a power (1−β) of 0.8 and α=0.05. N was calculated using EPIDAT software (http://www.sergas.es/Saude-publica/EPIDAT-4-2). All treatments were given as a single bolus (jugular) 60 minutes after the collagenase injection for ICH induction. We have chosen the dose of 1 mg/kg of CM352 because it showed the best pharmacological efficacy in mice in previous in vitro and in vivo studies conducted by our group.^19^, ^20^

Animals without hemorrhage (n=1), with small hemorrhage (n=2), or with hemorrhage located far from the basal ganglia (n=1) were excluded from the study before treatment administration.

The experimental procedure was performed following several criteria derived from the STAIR (Stroke Therapy Academic Industry Roundtable) group guidelines for preclinical evaluation of stroke therapeutics^22^: (1) ICH hematoma was evaluated at 1 hour, before treatment, by T2‐weighted magnetic resonance images (MRI) to confirm ICH, as an index of the reliability of the hemorrhagic model; (2) animals were randomly assigned to treatment groups of the study; (3) researchers were blinded to treatment administration; (4) researchers were blinded to treatments during outcome assessment; (5) temperature was controlled during the surgical period; and (6) a pharmacokinetics analysis of CM352 in rat blood samples was also conducted.

### Magnetic Resonance Imaging Protocol

Based on a previous hematoma growth profile study,^21^ hematoma volumes were assessed basally (1 hour after collagenase injection to induce ICH) and at 3 hours, 24 hours, and 14 days after ICH induction by means of magnetic resonance imaging conducted on a 9.4‐T horizontal bore magnet magnetic resonance imaging system (Biospec 94/20USR, Bruker BioSpin, Ettlingen, Germany) with 20‐cm‐wide actively shielded gradient coils (440 mT/m), as previously described.^21^ Radiofrequency transmission was achieved with a birdcage volume resonator, and signal was detected using a 4‐element surface coil positioned over the head of the animal. Gradient‐echo pilot scans were performed at the beginning of each imaging session for accurate positioning of the animal inside the magnet bore. Multislice axial T2‐weighted imaging was performed at baseline (1 hour after IHC induction), and at 3 hours, 24 hours, and 14 days after ICH. T2‐weighted images were acquired using a RARE (factor n=4) sequence with the following acquisition parameters: field‐of‐view 19.2×19.2 mm^2^, image matrix 192×192 (isotropic in‐plane resolution of 0.1×0.1 mm/pixel), 18 consecutive slices of 0.5 mm thickness.

Magnetic resonance data analysis was performed using Para Vision (Bruker Medical, Ettlingen, Germany), and images were processed with ImageJ (W. S. Rasband, National Institutes of Health, Bethesda, MD: http://rsb.info.nih.gov/ij). The analyzed region of interest was the hematoma and lesion region. Hematoma volumes (basal and 3 and 24 hours) as well as lesion volume at day 14 were manually traced from T2 maps, and the mean signal intensity was measured by 2 well‐trained imaging analysts blind to the experimental conditions.

### Behavioral Tests

Following STAIR criteria, animal models must show neurological and functional deficits in line with the produced lesion. The model of collagenase‐induced hemorrhage primarily damages the striatum, producing a small forelimb paresis contralateral to the lesion. Sensorimotor deficits after ischemic insult were assessed using the cylinder test, as previously described.^23^ This test evaluates limb asymmetry during the exploratory activity. Each animal was put in a cylinder with a 20‐cm‐diameter transparent base. A video camera was located under this transparent cylinder for recording the vertical exploratory movements of the animal with its anterior limbs for 2 to 10 minutes, depending on movement grade. For image recording and analysis, VirtulDub software was used. Analyzed behaviors were the numbers of times that the animal touched the cylinder wall and independent use of each limb in contact with the cylinder wall in each upward movement. The laterality index was calculated (the number of times that the animal touched the cylinder with the right leg during the ascendant movement by the number of times that the animals touched with each leg). This index is close to 0.5 for healthy animals and tends to 0 or 1 for animals that have a preferential use of the left or the right paw, respectively.

On the other hand, neurological deficit was evaluated with the Bederson scale,^24^ ranging from 0 (asymptomatic) to 8 (severe deficit), which included the following items: spontaneous movement, spontaneous rotation, spontaneous flexion of the contralateral forelimb, edge detection, turn after tail suspension, reflection of protection.

Behavioral studies were performed at baseline (before surgery) and at 24 hours and 14 days after ICH during the darkness cycle. An experienced investigator, blind to treatment and neuroimaging data, analyzed the behavioral tests.

### Blood Samples

Blood samples (0.5 mL) were drawn from the tail vein before surgery, immediately after surgery, and at 1 hour, 3 hours, 24 hours, and 14 days. Citrate or EDTA plasma was obtained by centrifugation, and samples were stored at −80°C. Enzyme‐linked immunosorbent assay was used to measure interleukin‐6 (IL‐6, ref. KRC0061, Life Technologies, Thermo Fisher Scientific, Waltham, MA), cellular fibronectin (ref. MBS007311, MyBioSource, San Diego, CA), and thrombin‐antithrombin complex (Enzygnost TAT, ref. OWMG15, Siemens Healthcare, Erlangen, Germany) in plasma, as biomarkers of inflammation, cellular damage, and hypercoagulability, respectively. The intra‐assay and interassay coefficients of variation for all molecular markers were <10%.

Prothrombin time (PT) and activated partial thromboplastin time (aPTT) were measured with STA‐Neoplastin R and PTT reagents (STA‐Compact, Stago, Barcelona, Spain). PT results were expressed as percentage of the standard value, and aPTT results were expressed in seconds.

### Animal Euthanasia and Brain Cryopreservation

After the completion of the neuroimaging study, 14 days after ICH, animals were euthanized by an overdose of anesthetic (sevoflurane 8%) and perfused with PBS and 4% formaldehyde. The brain was removed, axially sliced (2 mm thick), fixed in 4% formaldehyde (4 hours), transferred to 20% sucrose in PBS (4°C), included in optimal cutting temperature compound (Sakura Finetek, Torrance, CA), and stored at −80°C. Slices were cut into 8‐μm‐thick slices using a cryostat (Sakura Finetek Europe BV, Leiden, The Netherlands).

### Terminal Deoxynucleotidyl Transferase dUTP Nick End Labeling and Immunofluorescence

Brain slices were dried at 38°C for 3 hours, and the optimal cutting temperature compound was then removed by washing the slices twice in PBS. Antigen retrieval was performed by immersion of slices in a thermal bath of citrate buffer (pH 6.0) for 20 minutes at 99°C. To detect apoptotic cells, a terminal deoxynucleotidyl transferase dUTP nick end labeling assay was performed. After antigen retrieval, slices were incubated for 30 minutes in a blocking solution containing 3% bovine serum albumin in 0.1 mol/L Tris buffer saline (pH 7.5). After blocking, slices were washed in PBS and incubated for 1 hour at 38°C with Fluorescent In Situ Cell Death Detection Kit mix (Roche, Basel, Switzerland). After terminal deoxynucleotidyl transferase dUTP nick end labeling incubation, slices were washed and incubated with Hoechst (MW615.99; Thermo Fisher Scientific, Waltham, MA) diluted 1:6000 in PBS for 10 minutes in darkness. Slices were mounted (Polysciences, Eppelheim, Germany) and photographed in a Leica DMI 6000 B microscope with Leica Application Suite Advance Fluorescence software, version 1.0.0 (LAS AF; Leica Microsystems, Wetzlar, Germany).

A quantitative analysis of terminal deoxynucleotidyl transferase dUTP nick end labeling was performed using 3 animals from each group. Photomicrographs were obtained from the perihematoma area. Replicate photomicrographs were obtained from 2 consecutive slices containing the central part of the injured region. Positive nuclei were counted manually.

### Statistical Analysis

Results were expressed as mean±standard deviation or median [quartiles] for the continuous variables depending on their normal distribution or not, respectively. Statistical analyses were made using Mann‐Whitney or Wilcoxon tests when appropriate. A *P*<0.05 was considered as statistically significant. The statistical analysis was conducted using PASW Statistics 18 for Mac (SPSS Inc, Chicago, IL).

## Results

**Figure 1:**
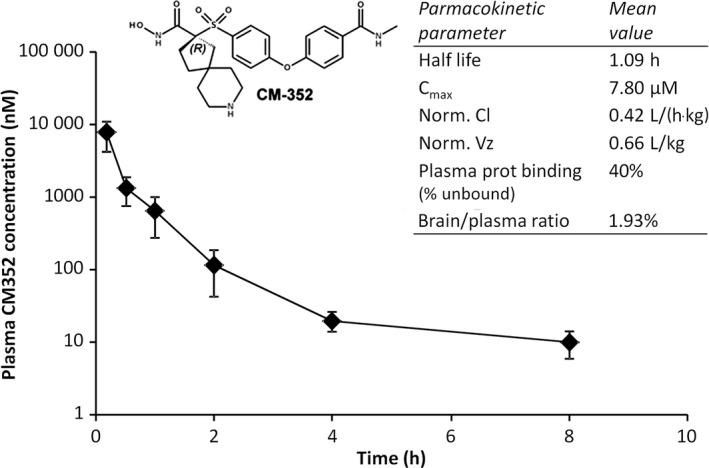
CM352 pharmacokinetics parameters estimated in rats after intravenous administration of 1 mg/kg (n=3). C_max_ indicates maximal plasma concentration; Norm Cl, normalized clearance; Norm Vz, normalized volume of distribution.

To determine the effect of the novel antifibrinolytic compound CM352 on hematoma growth in a rat model of collagenase‐induced ICH, we used it at the dose (1 mg/kg) previously reported to reduce hemorrhage in mouse tail and liver bleeding models.^20^ However, we first performed a detailed in vivo pharmacokinetic study in rats following intravenous administration of 1 mg/kg CM352 in order to validate results previously obtained in mice.^20^ As shown in Figure 1, CM352 reached a maximal plasma concentration of 7.8 μmol/L and showed low plasma protein binding and short half‐life (1.09 hours). Low volume of distribution and reduced brain/plasma ratio indicate that the drug remains mainly in the blood stream.

Since CM352 antifibrinolytic efficiency had been previously demonstrated in human and murine blood,^20^ we analyzed the effect of CM352 on tissue‐type plasminogen activator–induced fibrinolysis in rat whole blood. As expected, CM352 prolonged clot lysis time in the thromboelastometry assay, delaying fibrinolysis by 50% at a concentration of 3.7 μmol/L in rat blood, higher than the 0.2 μmol/L observed in mouse and 0.7 nmol/L found in man,^20^ without changes in coagulation activation and clot firmness.

Moreover, we analyzed CM352's effect on MMPs that had been reported to be upregulated after ICH in humans and experimental models.^14^, ^25^ We previously reported that CM352 efficiently inhibited MMP‐3 and MMP‐10,^20^ and in this study we describe that it also inhibits MMP‐2, MMP‐9, and MMP‐12 in the nanomolar range. However, GM6001, the broad‐range MMP inhibitor used as reference, was more efficient, particularly in the case of MMP‐7 (Table 1). By contrast, CM352 achieved 50% inhibition of MMP‐3 and MMP‐10 at a lower dose than GM6001. Because collagenase type VII was used to induce intracerebral hemorrhage, we tested CM352 and observed that it had no significant effect on collagenase activity in the 10 nmol/L to 10 μmol/L range of concentrations (IC50>10 μmol/L).

**Table 1:** MMP Inhibitory Activity (IC50) of CM352 and GM6001 (as Reference Compound)

	CM352	GM6001
MMP‐2	8.4 nmol/L	1.1 nmol/L
MMP‐3	15.0 nmol/L	20.7 nmol/L
MMP‐9	10.0 nmol/L	0.3 nmol/L
MMP‐7	16.0 μmol/L	9.0 nmol/L
MMP‐10	12.0 nmol/L	31.7 nmol/L
MMP‐12	7.6 nmol/L	1.2 nmol/L

MMP indicates matrix metalloproteinase.

### CM352 Reduces Hematoma Volume After ICH Induction

**Figure 2:**
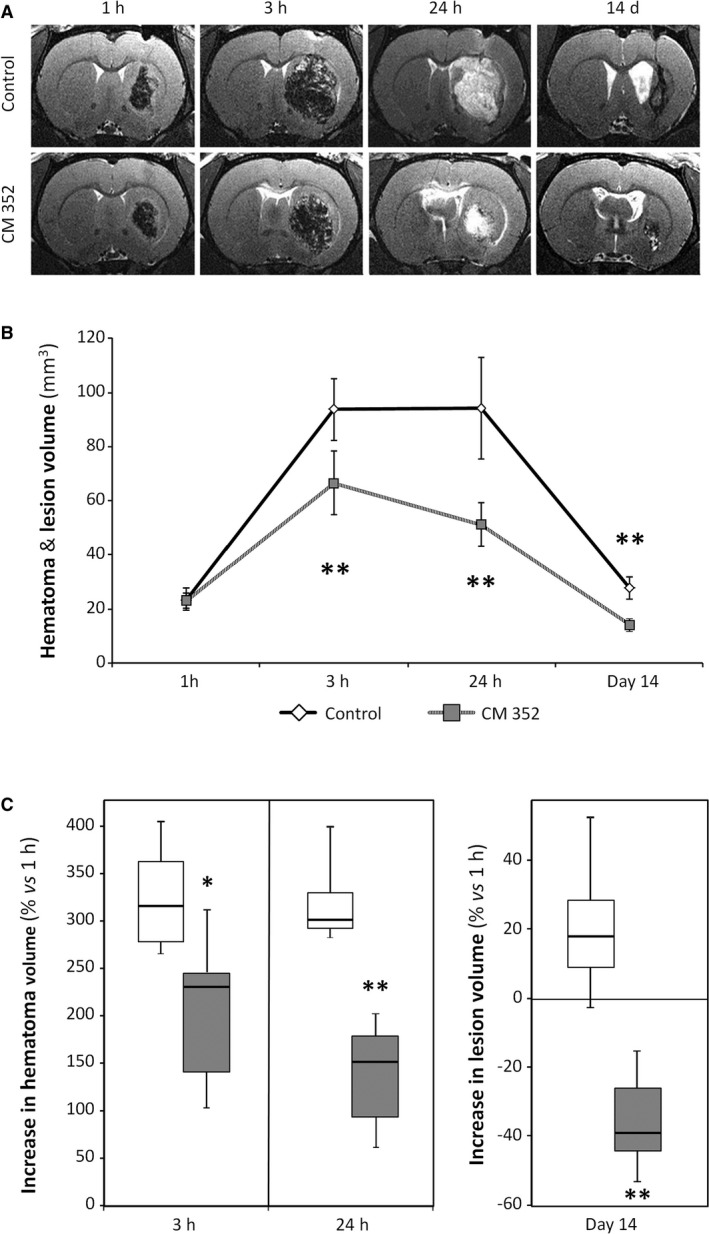
Early treatment with CM352 reduced hematoma after intraparenchymal injection of collagenase in rats. A, Analysis of hematoma volume was performed by T2‐weighted magnetic resonance image. Saline (n=6) or CM352 (1 mg/kg, n=6) was administered at 1 hour after collagenase injection. B, Hematoma volume was measured at 1 hour, 3 hours, 24 hours, and 14 days after collagenase injection. C, Analysis of hematoma expansion. Data are shown as mean±standard deviation or median [interquartile range]. Differences from control group are denoted as *P<0.05; **P<0.01. White boxes indicate saline controls; shaded boxes, CM352 treatment.

As shown in Figure 2A, intraparenchymal injection of collagenase caused an intracerebral hematoma with similar size in all animals at 1 hour, averaging 23.2±3.5 mm^3^ in rats randomized to CM352 and 22.8±3.1 mm^3^ in the control group. An expected increase in hematoma volume within 24 hours occurred in controls, but, interestingly, magnetic resonance imaging analysis showed a 29% reduction in hematoma volume for CM352‐treated rats compared with controls at 3 hours after ICH (*P*<0.01; Figure 2B). The difference from controls further increased up to 46% at 24 hours (*P*<0.01), the time of maximal hematoma volume. Analysis of hematoma expansion showed that CM352 treatment reduced it by 35% at 3 hours (310±62% versus 200±86%; *P*<0.05; Figure 2C) and by 56% at 24 hours (305±51% versus 133±63%; *P*<0.01; Figure 2C). Moreover, lesion volume at 14 days in the CM352 group was 48% smaller than that in controls (*P*<0.01) and even than the basal hematoma volume (*P*<0.01; Figure 2C).

### CM352 Improves Functional and Neurological Recovery

**Figure 3:**
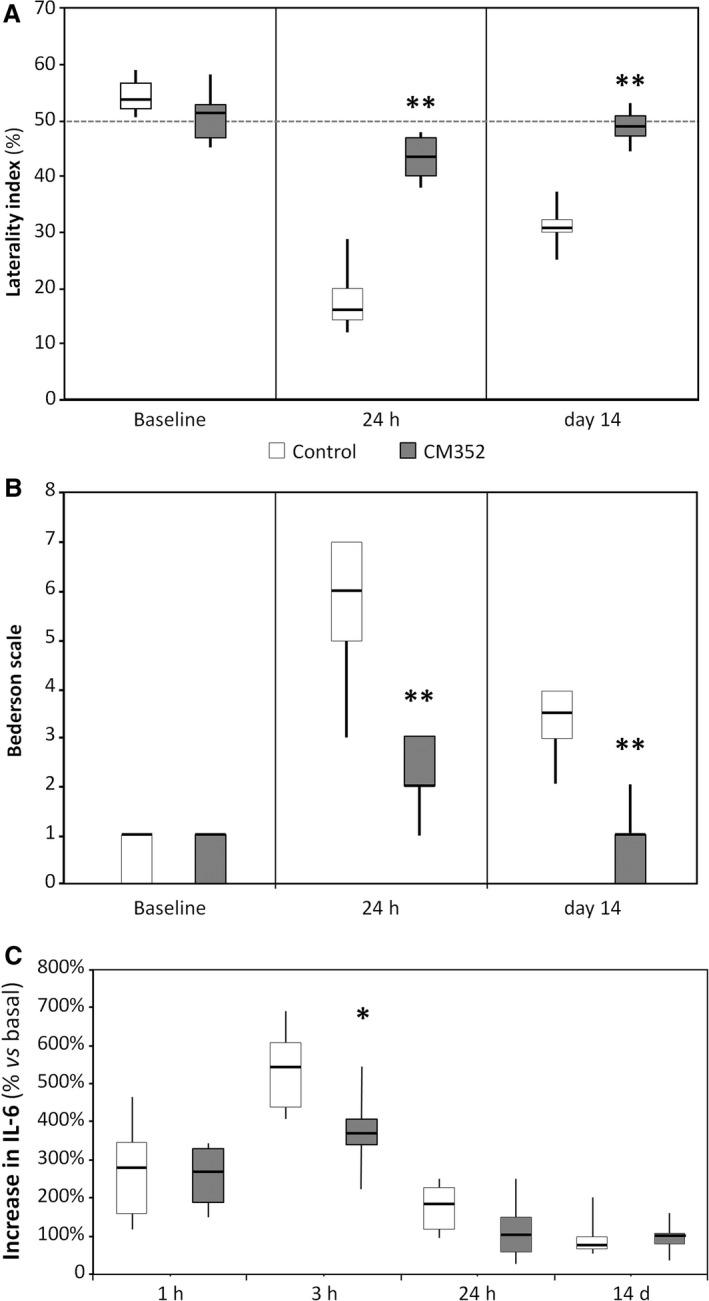
Early treatment with CM352 improved neurological and functional recovery after ICH. Functional deficit was evaluated by means of the laterality index (A) and neurological deficit by the Bederson scale (B). Neurologic tests were performed in control (n=6) and CM352‐treated rats (n=6) at 1 day before surgery (baseline) and at 24 hours and 14 days afterward. C, CM352 reduced circulating interleukin‐6 levels, a marker of systemic inflammation, after ICH (n=6). Data are shown as median [interquartile range]. Differences from control group are denoted as: **P<0.01; *P<0.05. ICH indicates intracerebral hemorrhage.

To determine whether the diminished hematoma expansion observed after CM352 administration could have any effect on the neurological and functional deficits caused by the hemorrhagic lesion, the Bederson scale and laterality test were performed before surgery (baseline) and at 24 hours and 14 days after ICH induction. Sensorimotor deficits were quantified using laterality index, which was close to 0.5 at baseline, before ICH for both experimental groups. Interestingly, as shown in Figure 3A, CM352 induced a better sensorimotor recovery at 24 hours and 14 days compared with the control group (all *P*<0.01).

Moreover, neurological deficit was assessed using the Bederson scale, which was close to 0 at baseline, before ICH for both experimental groups. However, as shown in Figure 3B, CM352 induced a better neurological functional recovery at 24 hours compared with the control group (*P*<0.01), with this beneficial effect of CM352 also being very noticeable at day 14 (*P*<0.01).

### Reduced Inflammation After ICH

Circulating markers of inflammation (IL‐6), blood‐brain barrier damage (cellular fibronectin) and hypercoagulability (thrombin‐antithrombin complex) were assessed after ICH in order to elucidate whether CM352 treatment could have an impact on these parameters. IL‐6 levels increased early after ICH, peaked at 3 hours, and then lowered steadily down to basal values by day 14 (Figure 3C). In spite of displaying similar IL‐6 concentration at baseline (11.7 [7.6‐13.9] versus 14.4 [11.4‐18.7] pg/mL), CM352‐treated animals exhibited a significant reduction at 3 hours (*P*<0.05). By contrast, no differences were found for cellular fibronectin and thrombin‐antithrombin complex levels between control and CM352 groups (data not shown).

### Terminal Deoxynucleotidyl Transferase dUTP Nick End Labeling Analysis

**Figure 4:**
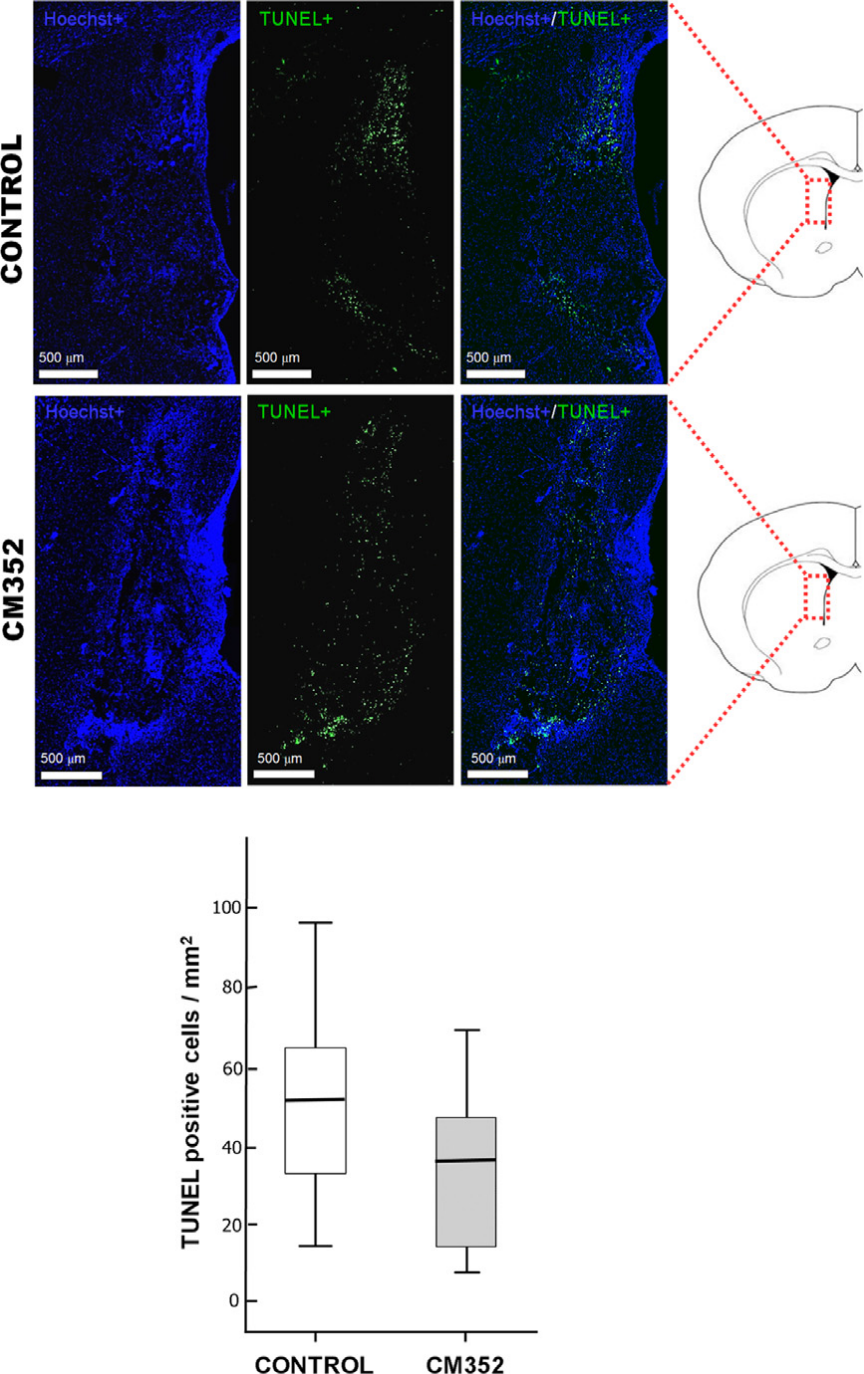
Analysis of apoptotic cells (TUNEL‐positive cells, in green) and nucleus (Hoechst marker, in blue) in brain samples of the control (n=3) and CM352‐treated rats (n=3) at 14 days after ICH. Apoptotic cell density in perihematomal area of the control and CM352 groups. Scale bars: 500 μm. Data are shown as median [interquartile range]. ICH indicates intracerebral hemorrhage; TUNEL, terminal deoxynucleotidyl transferase dUTP nick end labeling.

A histological analysis performed at 14 days after ICH revealed areas of terminal deoxynucleotidyl transferase dUTP nick end labeling–positive (dead or dying) cells in the perihematomal area in both control and CM352 groups (Figure 4). No effects on apoptosis in the perihematomal area were observed for CM352 treatment compared to the control group (Figure 4).

### Late CM352 Administration Reduces Brain Damage After ICH

**Figure 5:**
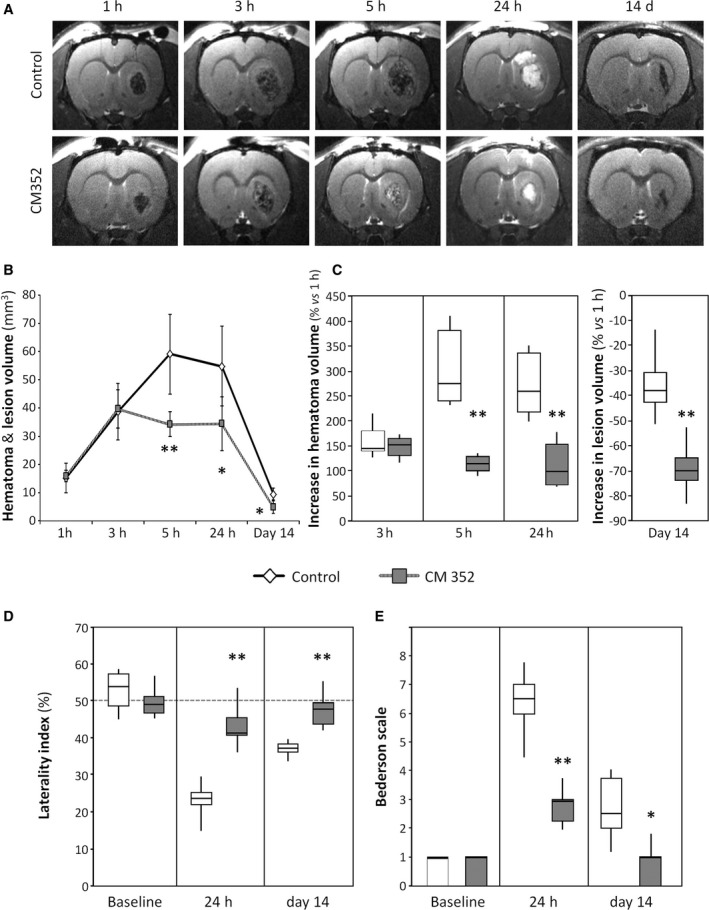
Late administration of CM352 reduced brain damage after intraparenchymal injection of collagenase in rats. A, Analysis of hematoma volume was performed by T2‐weighted magnetic resonance image. Saline (n=6) or CM352 (1 mg/kg, n=6) was administered 3 hours after collagenase injection. B, Hematoma volume was measured at 1 hour, 3 hours, 5 hours, 24 hours, and 14 days after collagenase injection. C, Analysis of hematoma expansion. Functional deficit was evaluated by means of the laterality index (D), and neurological deficit by the Bederson scale (E). Neurologic tests were performed in control (n=6) and CM352‐treated rats (n=6) at 1 day before surgery (baseline), at 24 hours, and 14 days afterward. Data are shown as mean±standard deviation or median [interquartile range]. Differences with control group are denoted as *P<0.05; **P<0.01.

Late (3 hours) administration of CM352 was tested in the same experimental model of ICH to better define its therapeutic effects. Intraparenchymal injection of collagenase caused an intracerebral hematoma (Figure 5A) with similar size in all animals at 1 hour (15.3±5.2 mm^3^ control versus 16.0±1.9 mm^3^ CM352) that increased in size by 150% at 3 hours (38.8±9.9 versus 39.8±6.7 mm^3^, respectively). The hematoma stabilized after CM352 administration at 3 hours, in stark contrast with the control group (*P*<0.01; Figure 5B), and differences in lesion size were patent between the groups up to day 14 (*P*<0.05). Analysis of hematoma expansion showed that CM352 treatment reduced it by 62% at 5 hours (306±82% versus 115±17%; *P*<0.01; Figure 5C) and by 58% at 24 hours (274±69% versus 114±50%; *P*<0.01; Figure 5C). Accordingly (Figure 5D), CM352 induced a better sensorimotor recovery at 24 hours and 14 days compared with the control group (all *P*<0.01). The Bederson scale also showed a better neurological function recovery at 24 hours (*P*<0.01) and at day 14 (*P*<0.05; Figure 5E).

PT and aPTT were measured in plasma samples to assess for potential effects of CM352 on extrinsic, intrinsic, or common coagulation pathways. No differences in PT or aPTT were observed between rats treated with CM352 (n=3) and controls (n=3) at any time point (Table 2).

**Table 2:** CM352 Does Not Alter Hemostasis After ICH

	Basal	3 H	5 H	24 H
PT, %				
Control	62.7±7.3	62.7±3.6	56.3±3.6	62.7±3.6
CM352	62.7±5.1	62.0±2.6	63.7±4.0	65.1±7.6
aPTT, s				
Control	19.0±1.0	19.7±1.5	19.0±3.5	19.0±2.6
CM352	21.0±4.0	21.7±4.5	21.7±4.0	21.7±2.5

Prothrombin time (PT) is expressed as percentage of the standard value, and activated partial thromboplastin time (aPTT) is expressed in seconds. ICH indicates intracerebral hemorrhage.

## Discussion

This study reports for first time that a dual antifibrinolytic and antiproteolytic agent (CM352) can effectively reduce hematoma expansion and brain damage in a rat model of collagenase‐induced ICH when administered as early (1 hour) or late (3 hours) treatment. These positive effects on hematoma growth are associated with improved neurological and functional recovery.

Main therapeutic interventions after ICH have been directed to either preventing hematoma expansion or protecting the neurovascular unit from secondary brain injury. Strategies addressing hematoma expansion by lowering blood pressure,^7^ surgical hematoma removal,^8^ reversal of anticoagulants,^3^ or hemostatic therapy with rFVIIa^9^ have provided evidence of reduced hematoma growth, but results regarding long‐term therapeutic benefit have been mainly disappointing.^4^ Moreover, to our knowledge, only a few preclinical studies have been conducted to assess the effects of the pharmacological modulation of hematoma growth.^6^ On the other hand, MMPs have been implicated in brain injury after ICH in animal models and in humans^12^, ^13^, ^26^; in particular, blood MMP‐9 concentration has been associated with hemorrhagic transformation.^27^ Rapid MMP upregulation after ICH has been proposed to cause neurovascular matrix degradation, proteolyzing the basal lamina around blood vessels and disrupting endothelial tight junctions, leading to edema, hemorrhage, and cell death.^13^ Based on this rationale, early treatment with broad‐range MMP inhibitors has been used in experimental models to reduce brain damage after ICH.^13^, ^14^

CM352 is a fast‐acting short‐lived new antifibrinolytic and broad‐range MMP inhibitor that has been demonstrated to be a very efficient antihemorrhagic agent without interfering with normal hemostatic function.^20^ We have shown that acute treatment with CM352 after ICH effectively reduces hematoma volume by 55% at 24 hours, which almost doubles the 30% reduction observed for rFVIIa in a similar model.^28^ Pharmacokinetic analysis demonstrates that at the therapeutic dose, in spite of its fast clearance rate, CM352 reaches plasma concentration in the micromolar range, as required for fibrinolysis inhibition ex vivo in the thromboelastometric assay with rat whole blood. These results are in accordance with the potent antihemorrhagic activity we had previously demonstrated in murine models of mild and severe bleeding.^20^ Moreover, reduction in hematoma expansion achieved with CM352 was accompanied by a substantial improvement in functional and neurological recovery, in marked contrast with clinical trials of rFVIIa, which reduced hematoma growth but did not improve survival or functional outcome after ICH.^9^ The use of the antifibrinolytic agents ε‐aminocaproic acid and TXA has been proposed after ICH (STOP‐AUST trial)^11^; however, the only experimental evidence available shows that TXA exacerbates edema in a mouse model of warfarin‐associated ICH.^29^ Moreover, in contrast with the potential risk for thrombotic events associated with treatment with rFVIIa, ε‐aminocaproic acid, or TXA, we have shown that CM352 does not alter hemostasis.^20^

CM352 is a dual antifibrinolytic/antiproteolytic agent, and we cannot explain reduced brain damage and improved functional and neurological outcome only through fibrinolysis inhibition. In fact, similar positive results have been obtained after late (3 hours) CM352 administration, when most of the effects could be attributed to prevention of MMP‐induced damage. MMP inhibition may offer an additional benefit in early stages because MMPs are upregulated after ICH and have been involved in secondary brain injury.^30^ CM352 may afford a degree of neuroprotection through MMP inhibition because we have shown that this drug efficiently inhibits most MMPs, which are upregulated after ICH.^14^ Moreover, because pharmacokinetic analysis shows that CM352 remains in blood above 10 nmol/L for 8 hours, it could be assumed that it could be actively inhibiting MMPs in this range of time after ICH.

On the other hand, increased IL‐6 levels had been associated with early hematoma growth in humans,^31^ and, accordingly, the reduction in this systemic inflammatory marker observed after CM352 treatment may be the result of the diminished bleeding but also a consequence of MMP inhibition. Interestingly, it had been previously shown, in a collagenase‐induced mouse model of ICH, that acute treatment with 100 mg/kg GM6001 (intraperitoneally for 48 hours), but not with 1 mg/kg, reduced leukocyte infiltration, oxidative stress, brain edema, and neuronal degeneration in association with a decrease in lesion volume and improved neurological outcome.^13^ This benefit had been explained mainly through preservation of the neurovascular matrix. However, using a mouse model of tail bleeding, we have recently shown that GM6001 at lower dose (1 mg/kg) has antihemorrhagic efficiency comparable to TXA (300 mg/kg), and CM352 is a more potent agent even at much lower doses (0.01 mg/kg).^20^ It could also be hypothesized that CM352 is inhibiting other proteases or cell‐death pathways involved in brain injury.^6^ However, previous off‐target selectivity profiling by biochemical characterization against a wide panel of metalloenzymes, including cathepsins, caspases, kalikreins, and neutrophil proteases, showed that CM352 had no significant effect on them.^20^ Thus, antihemorrhagic activity leading to reduction of intracerebral bleeding, combined with MMP inhibition, can explain observed benefits of CM352 treatment after ICH. At this point it is noteworthy that antifibrinolytic activity cannot be assumed for all MMP inhibitors but needs to be tested experimentally.

In the normal central nervous system MMPs are expressed at the low levels required for physiological functions. Early (24 hours) broad‐range MMP inhibition has been shown to reduce infarct size after focal cerebral ischemia, but delayed MMP inhibition (7 days) was associated with increased infarct volume.^32^ In this context intravenous administration and the short half‐life of CM352 allow precise administration of the compound and offer an additional benefit by sparing beneficial MMP functions in later stages of tissue remodeling and regeneration after ICH.

Finally, we have tested if CM352 could reduce the apoptotic cells in the perihematomal area because it has been suggested that apoptosis represents a prominent form of cell death associated with ICH in the perihematoma region.^33^ However, no effects on apoptosis in the perihematomal area were observed for CM352 treatment compared to the control group. This may be because we measured apoptosis at day 14, whereas the maximum apoptosis is described at day 5 after ICH.^33^

This study has a number of limitations. It has been designed to assess differences in hematoma volume, the main objective of the study, but it lacks the statistical power required to detect differences in biomarkers of brain or blood‐brain barrier damage because of the higher variability found in these parameters. In this model, collagenase injection breaks down small vessels and causes hemorrhage, mimicking spontaneous intraparenchymal bleeding in humans and hematoma expansion that occurs naturally in ICH patients. Moreover, we have used healthy animals, and the effectivity of CM352 should be confirmed in animal models including common ICH comorbidities such as hypertension and anticoagulants. Furthermore, future studies should also be conducted to test this new therapeutic approach in other related clinical settings where bleeding and neurovascular matrix degradation are relevant, such as hemorrhagic transformation of ischemic stroke after thrombolytic treatment or traumatic brain injury, especially in light of recent reports demonstrating the relevance of endogenous fibrinolysis after traumatic brain injury.^10^

In conclusion, we have shown that acute short‐term treatment with CM352, a dual‐acting fibrinolysis and MMP inhibitor, limits brain damage after ICH by reducing hematoma expansion and attenuating early inflammation, leading to improved functional and neurological recovery. These results suggest that CM352 may be an ideal new strategy for treating ICH, warranting clinical trials.

## Sources of Funding

This study has been funded through the Foundation for Applied Medical Research, University of Navarra (Pamplona, Spain), Spanish Ministry of Economy and Competitiveness (SAF2014‐56336, PTQ‐12‐05641, PTQ‐14‐07320), Instituto de Salud Carlos III (grants PI13/00292, PI14/01152, PI14/01879, PI15/01807), CIBER Cardiovascular (CB16/11/00371) the Spanish Research Network on Cerebrovascular Diseases (RD12/0014), the Xunta de Galicia (Department of Education, GRC2014/027), Spanish Society of Thrombosis and Hemostasia, and European Fund for Economic and Regional Development. Furthermore, Sobrino (CP12/03121) and Campos (CP14/00154) are recipients of research contracts from the Miguel Servet Program of Instituto de Salud Carlos III. The funders had no role in the review design, data collection and analysis, decision to publish, or preparation of the manuscript.

## Disclosures

None.
